# Quality Properties of Dried Banana Slices with Carboxymethyl Cellulose Coating Ultrasonic Pretreatments

**DOI:** 10.3390/foods14223904

**Published:** 2025-11-14

**Authors:** Fereshteh Nadery Dehsheikh, Somayeh Taghian Dinani, Piotr Koczoń, Joanna Bryś, Tomasz Niemiec, Lenka Kouřimská

**Affiliations:** 1 Department of Food Science, Shahreza Branch, Islamic Azad University, Shahreza 8648146411, Iran; fereshte.naderi.ir@gmail.com (F.N.D.); taghian@iaush.ac.ir (S.T.D.); 2 Department of Microbiology, Nutrition and Dietetics, Czech University of Life Sciences Prague, Kamycka 129, 165 00 Praha-Suchdol, Czech Republic; kourimska@af.czu.cz; 3 Department of Chemistry, Faculty of Food Sciences, Warsaw University of Life Sciences, Nowoursynowska 159 C, 02-787 Warsaw, Poland; joanna_brys@sggw.edu.pl; 4 Department of Animal Breeding, Institute of Animal Sciences, Warsaw University of Life Sciences, Nowoursynowska 159 C, 02-787 Warsaw, Poland; tomasz_niemiec@sggw.edu.pl

**Keywords:** banana, power ultrasound, CMC coating, quality attributes, antioxidant activity

## Abstract

Dried banana slices can be nutritious snacks that meet consumers’ needs. However, preserving their color, texture, and antioxidant properties is challenging during convective drying. The new approach aimed to produce high-quality dried banana slices with higher antioxidant activity and lower browning. In this paper, the simultaneous application of ultrasound (at three levels: 0 W, 500 W, and 1000 W) and carboxymethyl cellulose (CMC) coating (the ratio of banana slice mass to the coating solution mass (BS:CS) at three levels: 1:2, 1:3, and 1:4) pretreatments, and their combined effects on various characteristics of the finally obtained dried banana slices were examined. The convective drying of banana slices was carried out at 80 °C and 3 m/s air velocity to achieve a consistent moisture content of roughly 10% (kg water/kg dry matter). As the power of ultrasound was increased from 0 W to 1000 W and with changing the BS:CS ratio from 1:2 to 1:4, the results demonstrated that the effective water diffusion coefficient (D_eff_), water absorption capacity (WAC), and antioxidant activity (AA) of the dried banana slices were enhanced; however, their browning index (BI) decreased. Consequently, prior to convective drying, CMC coating using an ultrasonic system can be used as a practical strategy to produce fruit chips with desirable qualitative and nutritional properties.

## 1. Introduction

Due to increasing awareness of the importance of healthy snacks, dried fruits are becoming increasingly popular. Considering the high nutritional value of bananas as a good source of fiber; protein; carbohydrates; vitamins B6 and C [1]; and various minerals such as Mg, Fe, Zn, P, and Ca [2], this fruit is suitable as a nutritious snack. Nowadays, the worldwide consumption of bananas (*Musa* spp.) is growing. Owing to the presence of different antioxidants, it is an effective fruit in preventing cancer and heart disease as well [2]. Drying is one of the most important and oldest post-harvesting processes to prepare healthy dried products and prevent further deterioration [3]. Banana chips have been produced using different novel techniques such as osmotic drying [4,5], microwave [6,7,8], and freeze-drying [9,10,11]. Even though the majority of these methods produce high-quality dried products, they are expensive to start and maintain. Convective drying is still a common, simple, and cost-effective drying technology today. Since convective drying often requires high temperatures and a long period of drying [12], and as a result, undesirable changes in color [13] and quality and decreased rehydration capacity occur in the dried product [14], more studies are being conducted in recent years to improve this limitation. Consequently, in this paper, complementary processes such as ultrasound [15] and coating [16] can be utilized to improve the quality of dehydrated foods and overcome the limitations of the convective drying method.

The use of ultrasound has gained wide popularity due to its positive effects on food processing and other industries [17,18]. Ultrasonics can be effectively employed for various purposes such as homogenization, milling, high shear mixing, pasteurization, extraction [19], and hydrolysis of biomass [20]. In different studies, ultrasound waves were used as a pretreatment before convective drying [21,22,23,24,25,26]. The employment of ultrasound waves can result in increased mass transfer of water during convective drying by affecting the structure of the sample, especially its porosity and microscopic channels [23,27].

It is important to highlight that ultrasonic pretreatment of samples alone or in a pure water environment results in a decrease in their nutritional and sensory properties, such as a drop in antioxidant activity [28] and an increase in the browning index [29]. The ultrasound and coating process in organic solutions like CMC can be used to solve these problems. CMC is a cellulose derivative that acts as a water-soluble polymer that is resistant to heat, hydrolysis, and oxidation [30]. CMC, due to its ability to form a barrier or protective layer against gas transport and limit oxidative reactions, can reduce unfavorable changes in food that occur during drying [31]. As a result, some researchers applied CMC as an edible coating in different dying methods [32,33].

Although previous papers have been performed on CMC coating and ultrasound in convective drying, their combined application effects on the quality and antioxidant properties have been rarely investigated [34,35]. Therefore, there could still be a valuable opportunity for further research in this field for the drying of various agricultural products like bananas.

In this study, coating and ultrasound processes were simultaneously used to enhance the quality attributes of dried banana slices, specifically aiming to reduce the browning index and preserve their antioxidant activity more effectively. Accordingly, the influence of ultrasonic power (in three levels of 0 (without ultrasonic power application), 500, and 1000 W) and BS:CS mass ratio (in three levels of 1:2, 1:3, and 1:4) and their interaction on the drying rate, D_eff_, water WAC, AA, and BI of dried banana slices were studied. In addition, the microscopic structure was observed by scanning electron microscopy (SEM) images, and spectra of dried banana slices were evaluated with Fourier-Transform Infrared (FT-IR).

## 2. Materials and Methods

### 2.1. Ultrasonic-Coating Pretreatment

**Figure 1:**
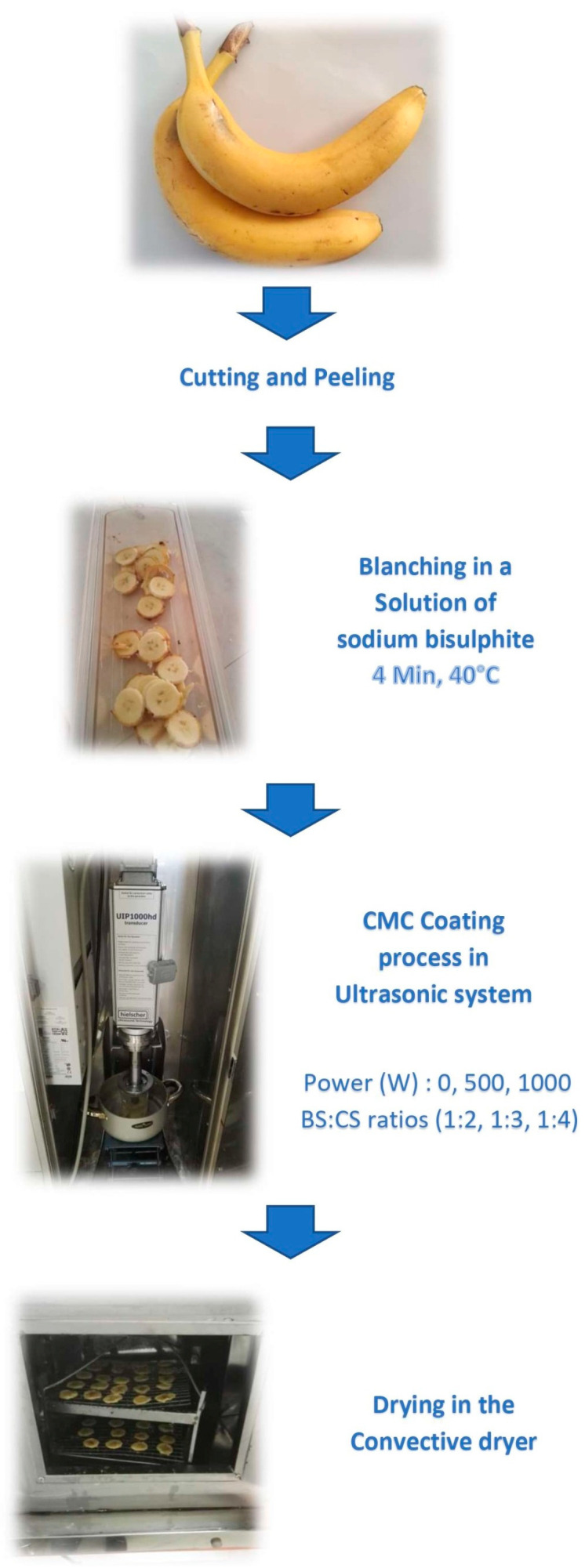
The experimental design.

Bananas (*Musa* spp.) with yellow peels were purchased from Shahin Shahr, Isfahan, Iran, and were washed with water. Then, they were manually peeled and cut into slices with a 5 mm thickness using a domestic slicing appliance (DL 610, Del Monti, Trieste, Italy). For 4 min at 40 °C, the slices were blanched in a solution of sodium bisulfite 0.25% (*w*/*w*) (Merck, Darmstadt, Germany) (the solution was prepared with distilled water, pH 7, and the temperature was checked by a thermometer placed in the solution) [36]. Three BS:CS ratios of 1:2, 1:3, and 1:4 for a constant weight of 300 g of the blanched banana slices with 1% CMC solution (Sigma-Aldrich, St. Louis, MI, USA) at volumes of 600, 900, and 1200 mL were prepared, respectively. The pH of the CMC solution was approximately 7. The coating process was performed for 30 min using an ultrasonic probe system (UIP 1000hd, Hielscher, Germany) at a frequency of 20 kHz with one of the three power stages of 0, 500, and 1000 W at a temperature of 45 ± 5 °C. To apply the ultrasonic process, the ultrasonic probe was placed approximately 2 cm inside the CMC solution with the banana slices. It should be mentioned that the beaker holding the samples with the CMC coating solution was put into a cold-water bath, and ice cubes were added to the water to control the temperature during the ultrasonic process. The temperature of the coating solution containing the banana slices was measured before and after the ultrasonic process with a thermometer. Following the coating process, the samples were extracted, and the convective was performed (Figure 1).

### 2.2. Convective Drying Method

To conduct the convective drying process, one layer of the pretreated banana slices (200 g) was put on two perforated metallic sheets (20 cm × 20 cm). Therefore, the initial mass distribution of banana slices on sheets was 0.25 g/cm^2^. Then, the sheets were placed in a convective dryer (Cabinet-Tunnel, Custom-built by Shahreza Azad University, Isfahan, Iran). The dryer was switched on 30 min before starting the drying process to reach the desired temperature (80 °C) [21,22,23] and airspeed (3 m/s) [37]. Throughout the drying process, the weight of the samples was recorded every quarter hour by a digital scale (Model JA, A&D, Tokyo, Japan, 0.0001 g). The slices were dried until their weight remained constant.

### 2.3. Analytical Methods

#### Moisture Content and Drying Rate

Moisture content (MC) of the sample slices in terms of kg water/kg dry matter was measured before entering the dryer and at the end of the convective drying process by the ALFA INC 55 model oven (Isfahan, Iran) method at approximately 70 °C [38]. The MC was calculated using Equation (1).

(1) $$\mathrm{MC}=\frac{W_{0}-W_{f}}{W_{f}}$$

This equation shows the initial and end weights of the banana samples in the oven as W_0_ and W_f_ (in grams), respectively. The dimensionless parameter of moisture ratio (MR) was determined by Equation (2).

(2) $$\mathrm{MR}=\frac{MC_{t}}{MC_{0}}$$

Equation (2) shows the moisture level of the banana slices at times t and at the initial time by MC_t_ and MC_0_ (water/kg dried matter), respectively. The drying rate (DR) was also obtained by Equation (3) in terms of kg water/(kg dry matter. min):

(3) $$\mathrm{DR}=\frac{MC_{t}-MC_{t+dt}}{\Delta t}$$

In this equation, the parameters MC_t_ and MC_t+dt_ (in kg water/kg dry matter) are the moisture contents at time t and t + dt, respectively, and Δt represents the considered length of time (min) [39]. In this study, at least three repetitions of all the tests were performed for each treatment.

### 2.4. Effective Water Diffusion Coefficient (D_eff_)

The effective water diffusion coefficient (D_eff_) is an important parameter associated with moisture transfer from inside the food to the surroundings [40]. D_eff_ can be calculated using Equation (4) (the mathematical simplification of Fick’s Second Law, assuming the thickness of the slice is small and comparable to its length).

(4) $$\mathrm{Ln}[\mathrm{MR}]=-\frac{\pi^{2}D_{eff}}{4L^{2}}t+\frac{8}{\pi^{2}}$$

In this equation, MR is the moisture ratio (described in Equation (2)), D_eff_ is the effective water diffusion coefficient (m^2^·s^−1^), L is the half-thickness of the banana slices when the mass transfer occurs on both sides of the slices (m), and t is the drying time (s). The slope of ln [MR] against time (−π^2^ D_eff_/4 L^2^) was used to calculate the D_eff_ parameter [38].

### 2.5. Water Absorption Capacity (WAC)

At the end of each drying treatment, approximately 7 g of the dried banana slices were placed in 500 mL of distilled water for 60 min at a temperature of 50 °C. After removing the samples from the distilled water, the samples were weighed. In the next step, the samples were dried in an oven (ALFA INC 55, Isfahan, Iran) at 70 °C [16] until their weight remained constant. Based on Equation (5), the WAC parameter was calculated.

(5) $$\mathrm{WAC}=\frac{W_{r}\left(100-S_{r}\right)-W_{d}\left(100-S_{d}\right)}{W_{0}\left(100-S_{0}\right)-W_{d}\left(100-S_{d}\right)}$$

In this equation, W is the weight (in grams); S is the dry matter content (%); and the subscripts r, 0, and d state the rehydrated, before drying, and dried conditions, respectively. The WHC varies from zero to one, and the lower values indicate the most damaged tissue in the samples [41]. Three WAC repetitions were carried out for each treatment.

### 2.6. Antioxidant Activity (AA)

To perform this test, the banana slices following the drying process were ground using an electric grinder (Bosch, TSM6A011W, Munich, Germany) at 25 °C. Then, the dried sample powder (1 g) was mixed with boiling distilled water (50 mL), and extraction at boiling temperature was performed for 45 min. Then, 2.7 mL ethanol (Sina Fariman Co., Khorasan, Iran) solution containing 2,2-diphenyl-1-picrylhydrazyl (DPPH) radicals (Sigma-Aldrich, St. Louis, MI, USA) with a concentration of 6 × 10^−5^ mol/L was mixed with the filtered extract (0.3 mL) into a test tube. Next, the sample was shaken using a shaker incubator (KMC 65, Fan Azma Gostar, Tehran, Iran) at 200 rpm for 15 min, and then it was placed in a dark place for one hour. Then, the absorbance of the extract was measured using a UV-Vis spectrophotometer (Model of Rayleigh-UV 9200, Beijing, China) at 517 nm. Finally, the antioxidant activity of the sample was calculated as the % inhibition of DPPH according to Equation (6) [15].

(6) $$\mathrm{Antioxidant}\;\mathrm{activity}\left(\%\;\mathrm{inhibition}\;\mathrm{of}\;\mathrm{DPPH}\right)=\frac{\left(A_{Control}-A_{Test}\right)\times 100}{A_{Test}}$$

In this equation, the A_Control_ and A_Test_ parameters are the absorption of the solutions without and with the extracts, respectively.

### 2.7. Browning Index (BI)

To measure BI, the L, a, and b color parameters of the surface of at least 5 banana slices before undergoing drying and after completion were measured by a colorimeter (Model TES-135A, Taipei, Taiwan). It should be noted that the L color indicator represents the range from brightness (100) to blackness (0). The ‘a’ color indicator is related to the redness (+a) to the greenness (−a), and the ‘b’ color indicator displays the yellowness (+b) to blueness (−b) [42]. Eventually, BI, which shows the intensity of the brown color, was determined using Equation (7) [43].

(7) $$\mathrm{BI}=\left[\frac{100-\left(X-0.31\right)}{0.17}\right]$$

(8) $$X=\frac{\left(a+1.75L\right)}{\left(5.64L+a-3.012b\right)}$$

To clarify, the parameter X in Equation (7) is calculated by Equation (8) [44].

### 2.8. Scanning Electron Microscopy (SEM) Images

In this study, the SEM images of the surface of the banana slices after drying were prepared by a scanning electron microscope (Model LEO435VP, Zeiss, Cambridge, UK) to investigate probable microscopic changes in the banana slices after drying. For this purpose, the dried banana slices’ surface was covered with a conductive material (a thin layer of gold) by the gold sputter coating machine (Model AGAR, Sputter Coater, Stansted, London, UK). Then, the SEM images of the surface were taken by the scanning electron microscope mentioned at a voltage of 18 kV.

### 2.9. Fourier-Transform Infrared (FT-IR) Spectroscopy

FT-IR spectroscopy was used to identify functional groups in the dried banana slices and the possible degradation of these groups due to the ultrasound and CMC coating processes. To carry out this test, approximately 2 mg of the dried banana slice powder and 200 mg of potassium bromide (Merck, Darmstadt, Germany) were mixed, and the obtained mixture was formed into a tablet. Then, the tablet was put in the FT-IR apparatus (Spectrum 65, PerkinElmer, Shelton, CT, USA). Finally, the sample’s FT-IR spectrum was obtained in an area of 4000–450 cm^−1^ with a spectral resolution of 4 cm^−1^ [45].

### 2.10. Statistical Analysis

In this study, each of the nine investigated treatments was repeated three times. The influence of two variables of ultrasound power (at three levels of 0, 500, and 1000 W) and the BS:CS ratio (at three levels of 1:2, 1:3, and 1:4), and the interaction of these two variables on dependent variables, was investigated by a Randomized Factorial Statistical design. The SPSS software version 19 and Duncan test were used to analyze the data, and *p* ≤ 0.05 was defined as a significant statistical value. The results were reported as mean ± standard error (SE).

## 3. Results and Discussion

### 3.1. Drying Kinetics

**Figure 2:**
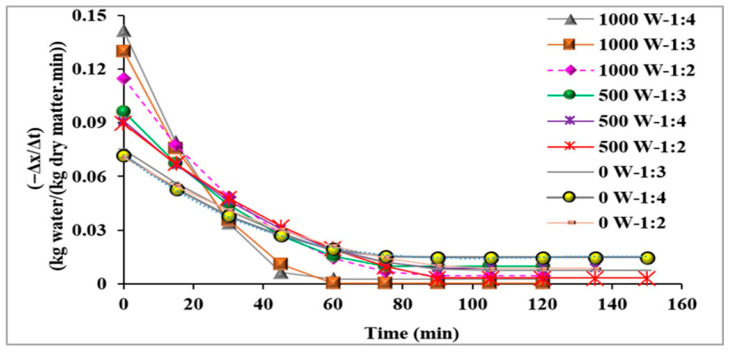
Drying rate versus time for banana slices.

Figure 2 illustrates the drying rate of the banana slices over time for various ultrasonic power levels and BS:CS ratios. This figure also displays that all the investigated drying treatments with two ultrasonic power levels (500 and 1000 W), especially those with pretreatment at the ultrasonic power of 1000 W, had a higher drying curve slope. Therefore, these treatments had a higher drying speed, especially at the beginning of the drying process, than those without the ultrasonic process (ultrasonic power of 0 W). A higher ultrasonic power can create the cavitation phenomenon, causing structural changes, microscopic channels, and near-surface pores in the banana slices. This can allow water to move from inside the sample to its surface, and consequently lead to faster water evaporation from the sample [46]. For that reason, the internal resistance to mass transfer declines with increasing ultrasound power [47], and thus, the drying rate improves efficiency.

As shown in Figure 2, the change in the BS:CS ratio did not affect drying time and rate of the banana slices. This result could be owing to the combined barrier and hydrophilic impacts of CMC coating on banana slices [48]. It is also possible that at all three levels of the BS:CS ratio, there is no significant difference in the amount of blockage of the capillary tubes and the porosities of samples caused by ultrasonication [49,50].

### 3.2. Effective Water Diffusion Coefficient (D_eff_)

**Figure 3:**
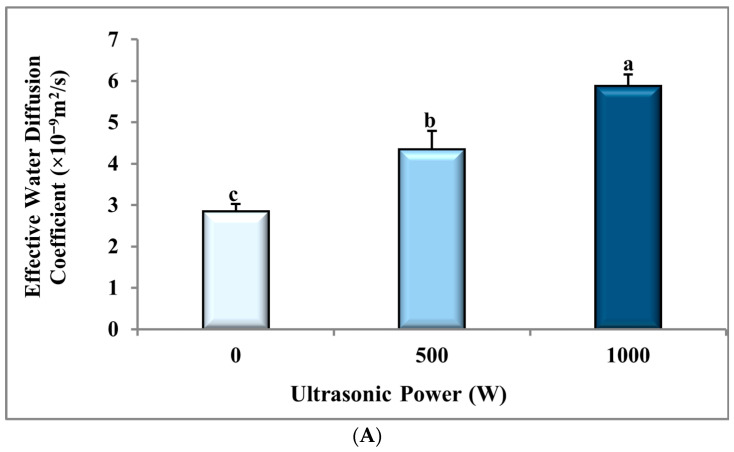

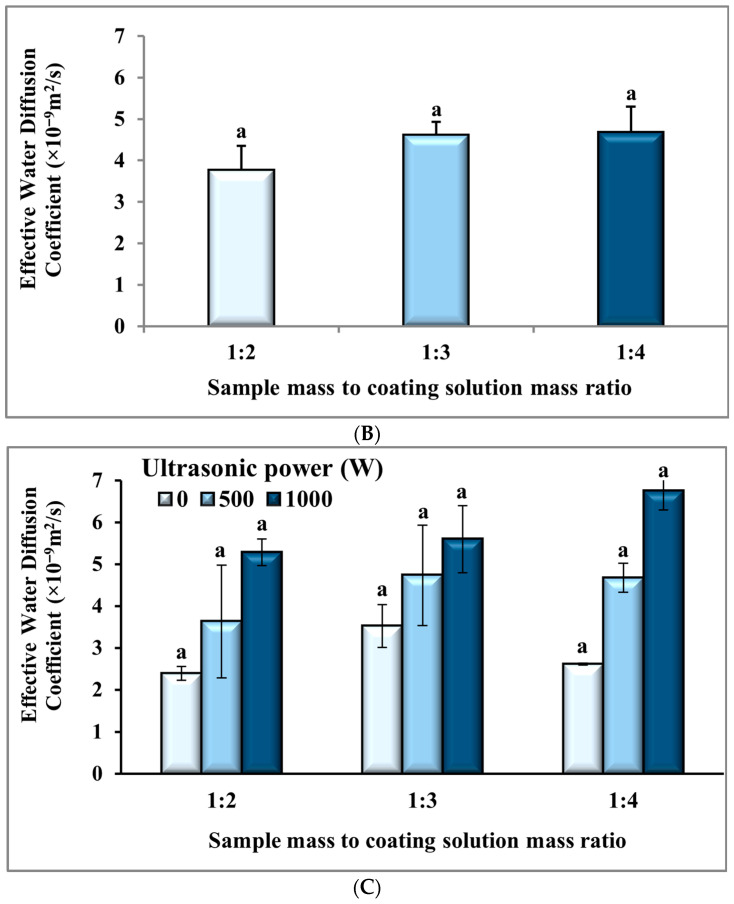
The effect of (**A**) ultrasonic power, (**B**) sample mass to coating solution mass ratio (BS:CS), and (**C**) their interaction effect on the effective water diffusion coefficient of the dried banana slices. Different English letters represent a statistically significant difference.

The ANOVA analysis (Table 1) displays that the effect of ultrasonic power (*p* ≤ 0.001) on the D_eff_ of the dried banana slices had a statistically significant impact. In contrast, the BS:CS ratio and the interaction of the ultrasonic power and the BS:CS ratio did not show a statistically significant influence (*p* > 0.05). As the power of ultrasound increased from 0 W to 1000 W, the D_eff_ of drying the banana samples significantly improved from (2.84 ± 0.19) × 10^−10^ m^2^/s to (5.87 ± 0.28) × 10^−10^ m^2^/s, which is equivalent to a 106.70% increase (*p* ≤ 0.001), as shown in Figure 3A and Table 2. This enhancement is likely due to structural changes in the tissue of samples that were pretreated by ultrasonic power, which can improve the mass transfer rate, D_eff_, and drying rate, and consequently decrease drying time [38]. Ortuño et al. (2010) [51] reported similar findings for the orange peel pretreated at an ultrasonic power of 90 W in comparison with control samples (without prior ultrasonic pretreatment). Figure 3B and Table 3 display that the D_eff_ of the banana slice drying insignificantly improved from (3.76 ± 0.58) × 10^−10^ m^2^/s to (4.67 ± 0.62) × 10^−10^ m^2^/s (a 24.20% increase) with a change in the BS:CS ratio from 1:2 to 1:4. This demonstrates that changing the BS:CS ratio did not have a significant influence on the D_eff_ of drying the banana slices, and therefore, on the rate and time of drying the banana slices (Figure 1). Figure 3C shows the interaction of the ultrasonic power and the BS:CS ratio on the D_eff_ parameter. This figure displays that the lowest mean of D_eff_ was equivalent to (2.39 ± 0.17) × 10^−10^ m^2^/s using the 0 W-1:2 pretreatment, while the highest mean (6.75 ± 0.47) × 10^−10^ m^2^/s was related to the 1000 W-1:4 pretreatment. Therefore, the D_eff_ parameter improvement can be observed with rising the ultrasonic power from 0 to 1000 W and a change in the BS:CS ratio from 1:2 to 1:4. The combined effect of the pretreatments on D_eff_ of the dried banana slices may be related to changes in the viscosity of the CMC solution, which is helpful in better uniformity and spread of the coating on the banana slices and cavitation efficiency [52]. The reduction in viscosity makes moisture transfer easier, and D_eff_ is increased.

### 3.3. Water Absorption Capacity (WAC)

**Figure 4:**
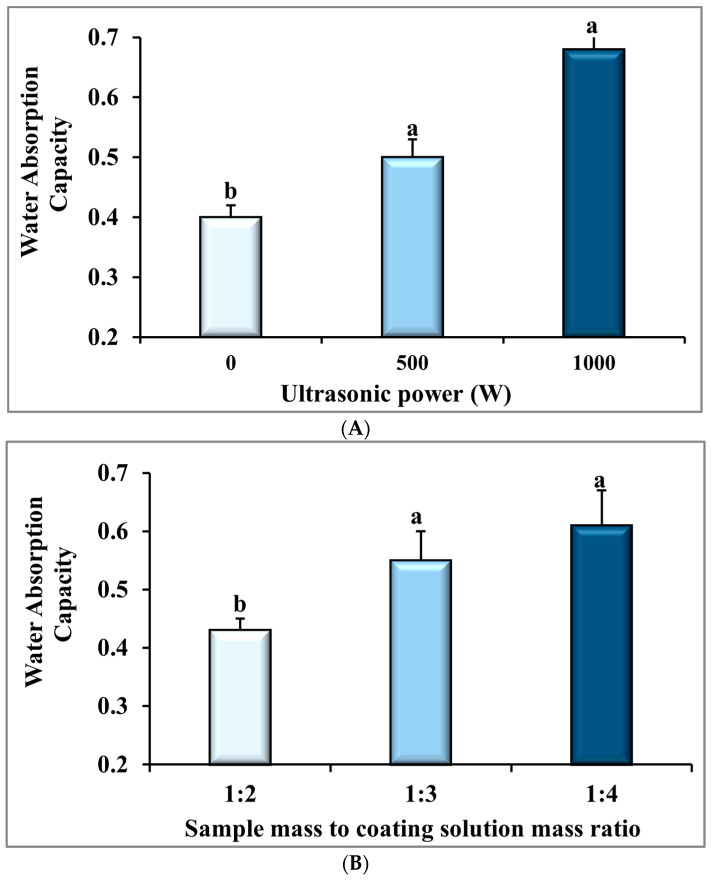

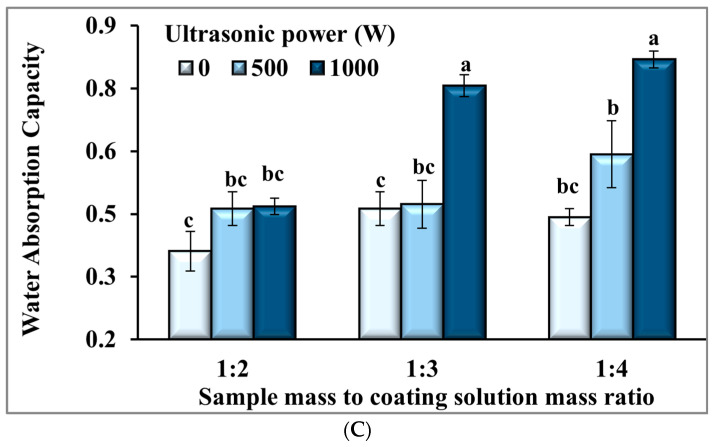
The effect of (**A**) ultrasonic power, (**B**) sample mass to coating solution mass ratio (BS:CS), and (**C**) their interaction effect on water absorption capacity of the dried banana slices. Different English letters represent a statistically significant difference.

The higher rehydration ability or WAC of dried samples is more appropriate and desirable for customers, and indicates less damage to the structure of dried samples [46]. The statistical results, presented in Table 1, show that the influence of ultrasonic power (*p* ≤ 0.001), the BS:CS ratio (*p* ≤ 0.01), and their interaction (*p* ≤ 0.05) on the WAC of the dried sample were significant. Based on Figure 4A and details in Table 2, increasing the ultrasonic power from 0 W to 1000 W led to a significant rise in the mean WAC from 0.41 ± 0.02 to 0.68 ± 0.05, which indicated a 65.85% growth (*p* ≤ 0.001). The WAC increase in the dried banana slices with a higher ultrasonic treatment can be attributed to the enhanced drying rate and the drying time decrease in this condition (Figure 2). This is due to the improved drying rate and shorter drying time, which help keep microscopic channels in the ultrasonically pretreated banana slices. As a result, there is greater porosity and more intercellular spaces, which leads to increased WAC [53,54,55]. Similar effects were reported for kiwi slices, with longer sonication times (10, 20, and 30 min) increasing WAC by shorter convective drying [56]. Jambrak et al. [46] examined the effects of different ultrasonic intensities on the rehydration ratio of convectively dried mushrooms, Brussels sprouts, and cauliflowers at a temperature of 60 °C and an airspeed of 0.3 m/s. These researchers reported a reduction in the drying time and an increase in the rehydration ratio of samples pretreated in an ultrasonic probe system with high intensity (39–43 W/cm^2^) compared to those pretreated in an ultrasonic bath system with low intensity (0.5 W/cm^2^), and compared to control samples without ultrasonic pretreatment. The researchers also noted that the increase in the rehydration ratio was due to the increase in porosity and lower sample density during drying, which is in agreement with our findings in this study.

The WAC of the dried samples rose considerably (*p* < 0.01) when the BS:CS ratio changed from 1:1 to 1:4, from 0.43 ± 0.02 to 0.61 ± 0.06, respectively (Figure 4B and Table 3). It is important to mention that there were no significant differences (*p* > 0.05) in the water activity of the dried banana samples with BS:CS ratios of 1:3 and 1:4. An increase in the WAC of the dried banana slices by changing the BS:CS ratio from 1:2 to 1:4 is probably related to the formation of an improved CMC layer with better protective function on the surface of the samples [16]. In these conditions, the CMC molecules are unlikely to penetrate the pores of the samples fully; therefore, the porous structure is preserved [49]. In other words, the coating on the sample surface could enhance water absorption by increasing hydrophilic points and water absorption on the sample surface [57]. Taghian Dinani et al. [16] reported that when a higher concentration of CMC solution was applied, the mean WAC of dried mushroom slices was improved. This was owing to the hydrophilic nature of CMC molecules on the dried mushroom slices. The results are similar to our results regarding the positive influence of an increase in the BS:CS ratio on obtaining a more uniform hydrocolloid film and the water absorption of samples.

Figure 4C presents the significant interaction of the ultrasonic power and the BS:CS ratio (*p* ≤ 0.05) on the WAC of the dried samples. This diagram shows that the highest and lowest mean WAC were obtained under the 1000 W-1:4 (0.81 ± 0.02) and 0 W-1:2 (0.36 ± 0.04) treatments, respectively. It is important to note that, according to Figure 2, the lowest and highest drying rates were related to the 0 W-1:2 and 1000 W-1:4 treatments, in the corresponding order. Hence, the interpretation associated with increasing WAC by reducing the drying time or decreasing WAC by increasing the drying time is confirmed. In agreement with our findings, Askari et al. [58] indicated that the surface coating of apple slices with starch, pectin, and CaCl_2_ contributed to the increased porosity, lower density, and increased rehydration capacity of the samples.

### 3.4. Antioxidant Activity (AA)

**Figure 5:**
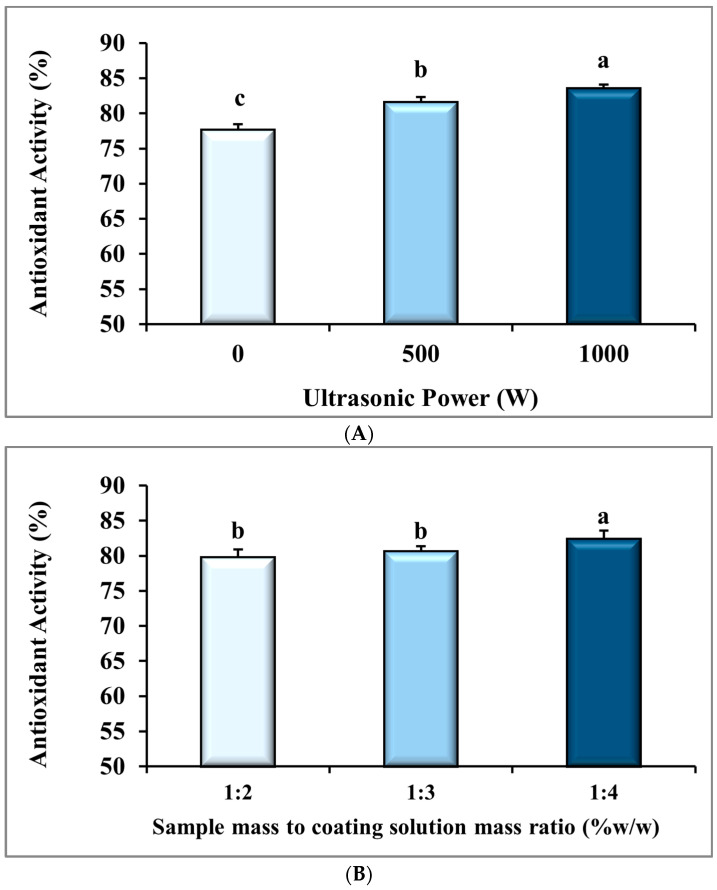

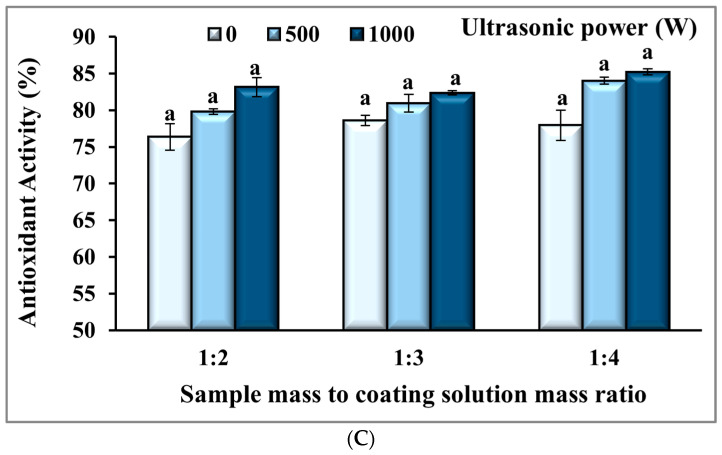
The effect of (**A**) ultrasonic power, (**B**) sample mass to coating solution mass ratio (BS:CS), and (**C**) their interaction effect on antioxidant activity (%) of the dried banana slices. Different English letters represent a statistically significant difference.

The ANOVA analysis in Table 1 revealed that the effects of the ultrasonic power (*p* ≤ 0.001) and the BS:CS ratio (*p* ≤ 0.05) on the AA of the dried banana slices were statistically significant. However, the impact of their interaction was non-significant (*p* > 0.05) on this response. Figure 5A and Table 2 show that the mean AA of the dried samples with an increase in the ultrasonic power from 0 W to 1000 W was significantly improved from 77.63 ± 0.89 to 83.57 ± 0.60, equivalent to 7.6% (*p* ≤ 0.001). This improvement is attributed to faster drying rates and shorter drying times (Figure 2), which reduced antioxidant degradation. Rodríguez et al. [26] reported more AA of apple cubes pretreated at an ultrasonic power of 30.8 kW/cm^3^, owing to their shorter drying time compared to those pretreated at 18.5 kW/cm^3^ or control samples without prior ultrasonic pretreatment.

Figure 5B and Table 3 display that the mean AA of the dried samples with a change in the BS:CS ratio from 1:2 to 1:4 significantly improved from 79.77 ± 1.18 to 82.39 ± 1.28 (*p* ≤ 0.05). The immersion of the samples in a higher volume of CMC solution provides a better coating layer on the sample surface [16]. A better coating layer on the sample surface reduces the heat and oxygen exposure of bioactive ingredients. In addition, the coating layer decreases gas exchange in the samples [59,60], which in turn leads to reducing the degradation of antioxidants and improving the AA of the dried samples. Lobo et al. [61] observed that the application of a higher concentration of CMC during the coating procedure helped preserve antioxidants in mango pulp. Taghian Dinani et al. [16] also reported that by increasing the concentration of CMC solution from 0 to 3%, the scavenging effect of dried mushroom slices rose by 85.02%. This improvement in the scavenging effect could be related to a more effective coating layer formed on the sample.

Figure 5C illustrates the interaction of the ultrasonic power and the BS:CS ratio on the AA parameter of the dried banana slices. Based on this figure, the lowest mean of AA was equivalent to 76.3 ± 1.8% using the pretreatment of 0 W-1:2, and the highest mean of this dependent variable reached 85.2 ± 0.4% with the 1000 W-1:4 pretreatment. The figure also presents the AA parameter improvement with an increase in ultrasonic power from 0 W to 1000 W and BS:CS ratio changes from 1:2 to 1:4. Nadery Dehsheikh and Taghian Dinani [62] conducted an investigation using an electrohydrodynamic system (EHD) and CMC solution before convective drying of banana slices. They reported that by increasing the EHD voltage from 0 to 20 kV and changing the CMC solution ratio from 1:1 to 1:2, the total phenolic content increased by 20.3% and 16.9%, respectively. A reduced drying time resulted in an increase in the total phenolic content and an improvement in nutritional qualities according to these researchers. Therefore, a uniform coating can help to better preserve the nutritional properties of foods [62]. According to Sakooei-Vayghan et al. [63], apricot cubes pretreated by ultrasound-assisted osmotic dehydration, osmotic dehydration, and coating (pectin + ascorbic acid) showed higher total antioxidant activity than control samples without pretreatment. The protective coating and the lower drying time were the reasons. Zang et al. [35] and An et al. [50] had also stated that pretreatment with ultrasound-CMC is effective in maintaining the antioxidant properties during drying of cherries and turmeric.

Since the antioxidant activity of fruits relies on their phenolic compounds [64], the amount of antioxidant activity can be linked to their nutritional properties. This is due to ultrasound-assisted release of phenolic compounds, which donate hydrogen and remove free radicals [65]. Meanwhile, osmotic pretreatment protects these compounds from damage caused by oxidation and heat [66]. That is, increasing the antioxidant properties of the samples might be considered to lead to better nutritional properties [67].

### 3.5. Browning Index (BI)

**Figure 6:**
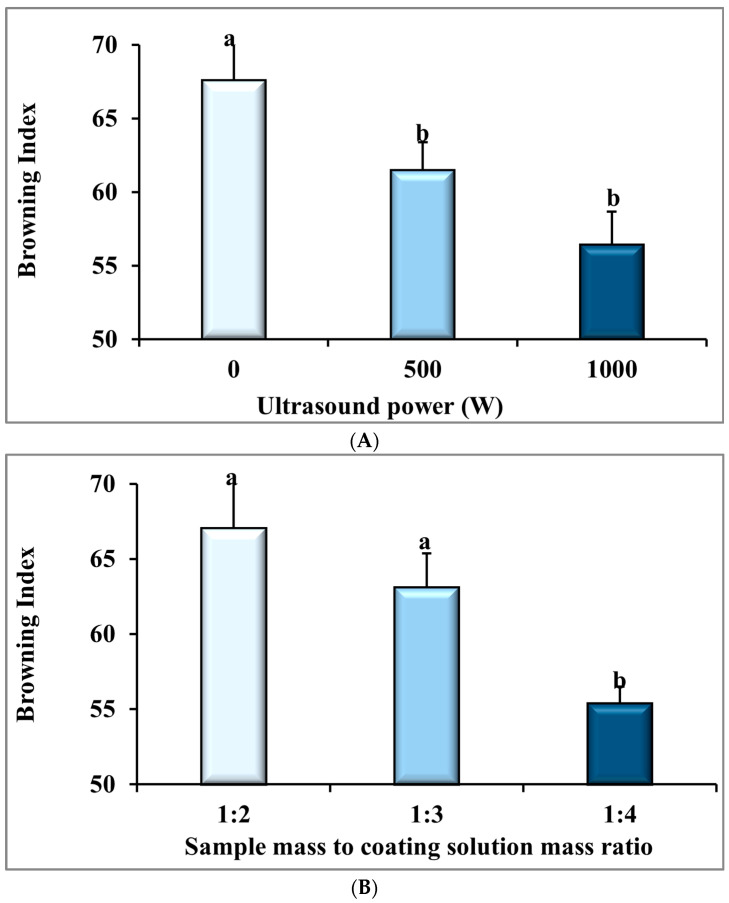

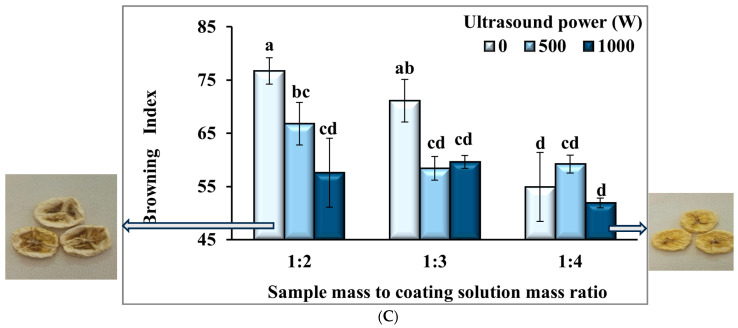
The effect of (**A**) ultrasonic power, (**B**) sample mass to coating solution mass ratio (BS:CS), and (**C**) their interaction effect on the browning index of the dried banana slices. Different English letters represent a statistically significant difference.

The ANOVA analysis in Table 1 shows that the ultrasonic power (*p* ≤ 0.01), the BS:CS ratio (*p* ≤ 0.01), and their interactions (*p* ≤ 0.05) had statistically significant effects on the BI of the dried banana slices. Figure 6A and Table 2 display that the mean BI of the dried banana slices significantly reduced from 67.59 ± 3.44 to 56.41 ± 2.26 with the ultrasonic power increase from 0 W to 1000 W, equal to 16.5% (*p* ≤ 0.01). This can be attributed to the rise in the drying rate (Figure 2) and the increase in the color parameter L or the brightness of the samples in these conditions (L color parameter data are not presented in this paper). It should be noted that the decomposition of carotenoids and the formation of brown pigments decrease with increasing drying rate and a shorter drying time, which results in a lower BI. Deng and Zhao [68] found that the ultrasonic process before the freeze-drying process decreased the browning of apple cubes due to the faster drying process.

With a change in the BS:CS ratio from 1:1 to 1:4, the mean BI of the dried banana slices declined significantly from 67.03 ± 3.62 to 55.37 ± 1.13, or 17.4% (*p* < 0.01), as shown in Figure 6B and Table 3. Therefore, a better CMC coating is produced when the samples are immersed in a larger volume of the CMC solution [16]. In addition, by reducing the gas exchange in the samples [60], oxidative reactions, both enzymatic and non-enzymatic, in the dried banana slices are reduced. As a result, the BI decreases. Nadery Dehsheikh and Taghian Dinani [62] observed that by raising the BS:CS ratio from 1:2 to 1:4, the total color changes in dried banana slices were reduced, which indicated a reduction in browning of the dried banana samples. Taghian Dinani et al. [16] also showed that increasing the CMC solution content from 0% to 3% reduced the brightness changes in dried mushroom slices by 40%. As a result, the browning index lowering of dried mushroom slices can be predicted.

Figure 6C shows the interaction of the power of ultrasound and the BS:CS ratio on the BI parameter. This figure displays that the highest observed value of BI was equivalent to 76.7 ± 2.5 using the pretreatment of 0 W-1:2 and the lowest value of this dependent variable was recorded 51.9 ± 0.9 for the pretreatment of 1000 W-1:4. This result is well seen in two pictures on the right and left sides of Figure 6C, which were taken from the dried banana slices of 1000 W-1:4 and 0 W-1:2 treatments, respectively. The photo on the right is presented to confirm lower browning and better quality of the dried banana slices pretreated with 1000 W-1:4 than those of the dried banana slices pretreated with 0 W-1:2 in the left photo. Garcia-Noguera et al. [69] investigated the use of two ultrasonic pretreatments and osmotic dehydration in freeze-dried strawberries. It has been reported in this study that, with the long ultrasonic process and high concentrations of sucrose, the color of the dried products became brighter. Biswas et al. [66] reported that the combined use of ultrasound and osmotic pretreatment created a synergistic effect that limited browning reactions and helped preserve antioxidant compounds by improving mass transfer and reducing oxidative damage during drying.

It is worth mentioning that the browning index and nutritional properties of dried samples are closely intertwined. In other words, when there are fewer non-enzymatic reactions in the sample, such as the Maillard reaction, then less browning index and more amino acids in the samples can be preserved [70]. In general, a decline in the BI parameter can be regarded as a better nutritional property of dried samples [71]. In our study, the negative correlation between the BI and AA parameters, as reported in Table 4, can confirm this conclusion. Similar results were obtained by Zang et al. [35].

### 3.6. Fourier-Transform Infrared Spectroscopy (FT-IR)

**Figure 7:**
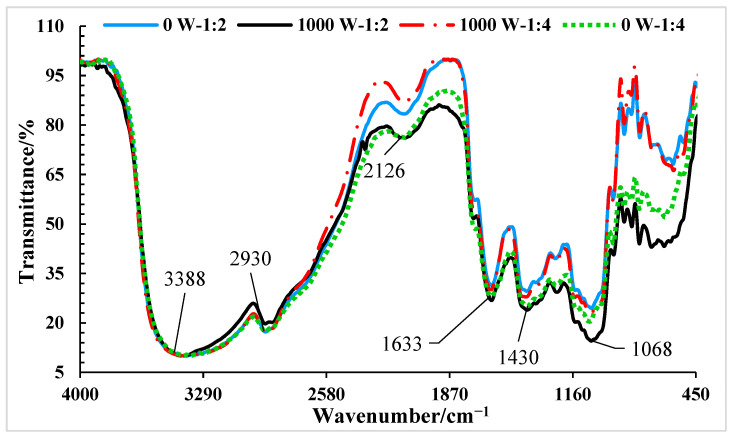
FT-IR spectra of dried banana slices pretreated with ultrasonic power of 0 W—sample mass to coating solution mass ratio of 1:2; ultrasonic power of 1000 W—sample mass to coating solution mass ratio of 1:2; ultrasonic power of 0 W—sample mass to coating solution mass ratio of 1:4; and ultrasonic power of 1000 W—sample mass to coating solution mass ratio of 1:4.

The FT-IR test was used to determine the effects of pretreatments of 0 W-1:2, 1000 W-1:2, 0 W-1:4, and 1000 W-1:4 on the functional groups of the banana slices after drying. In Figure 6, the absorption band at 3383 cm^−1^ indicates the alcohol/phenol OH stretch bond. The band at 2930 cm^−1^ also represents the alkyl CH stretch [72]. The band at 2126 cm^−1^ represents the C≡C symmetry stretching vibration, the band at 1633 cm^−1^ originates from the C=C aromatic stretching [73], and the band at 1633 cm^−1^ denotes the symmetric stretching of the C=O bond from the carboxyl group (-COOH) [74]. The band at 1068 cm^−1^ represents the C–OH stretching band, and the band at 628 cm^−1^ is assigned to the CH=CH stretching vibration of an aromatic ring [75]. Based on Figure 6, it can be argued that the FT-IR spectra of all four pretreatments contain the main bands previously mentioned. Moreover, the increased peak heights at 1430 and 1068 cm^−1^ showed enhanced C–H bending and C–O stretching vibrations that reflect improved coating and hydrogen bonding. The peaks at 2126 and 1633 cm^−1^ indicated slight changes in %Transmittance. This suggests minor alterations in the chemical environment due to ultrasound. Importantly, no shifts in band positions were seen, confirming that the basic chemical structure of the banana slices stayed the same [76]. Therefore, based on the results obtained from Figure 7, it can be interpreted that the application of two levels of the BS:CS ratio of 1:2 and 1:4, and even pretreatment with 1000 W ultrasonic power compared to the absence of ultrasound (ultrasonic power of 0 W), did not damage or change the functional groups of the banana slices during convective drying. Moreover, the outcomes supported the effective coating formation and enhanced molecular interactions. The outcomes of this study are consistent with the results reported by Khalili and Taghian Dinani [75] on the application of ultrasonic pretreatment at 10, 30, and 50 min in the extraction of phenolic compounds from the olive-waste cake. They reported that the IR spectrum of the extracts obtained by using ultrasounds showed no changes to the type of functional groups. The bands generated by those groups were at the same positions in the IR spectrum.

### 3.7. Scanning Electron Microscopy (SEM) Pictures

**Figure 8:**
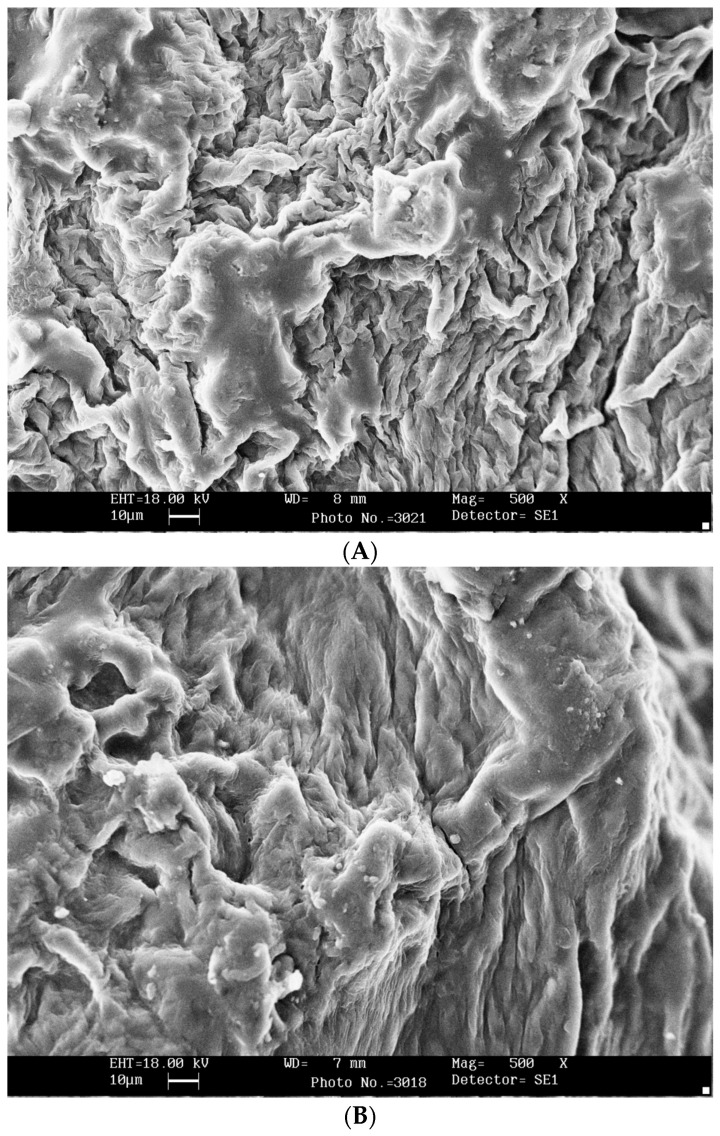

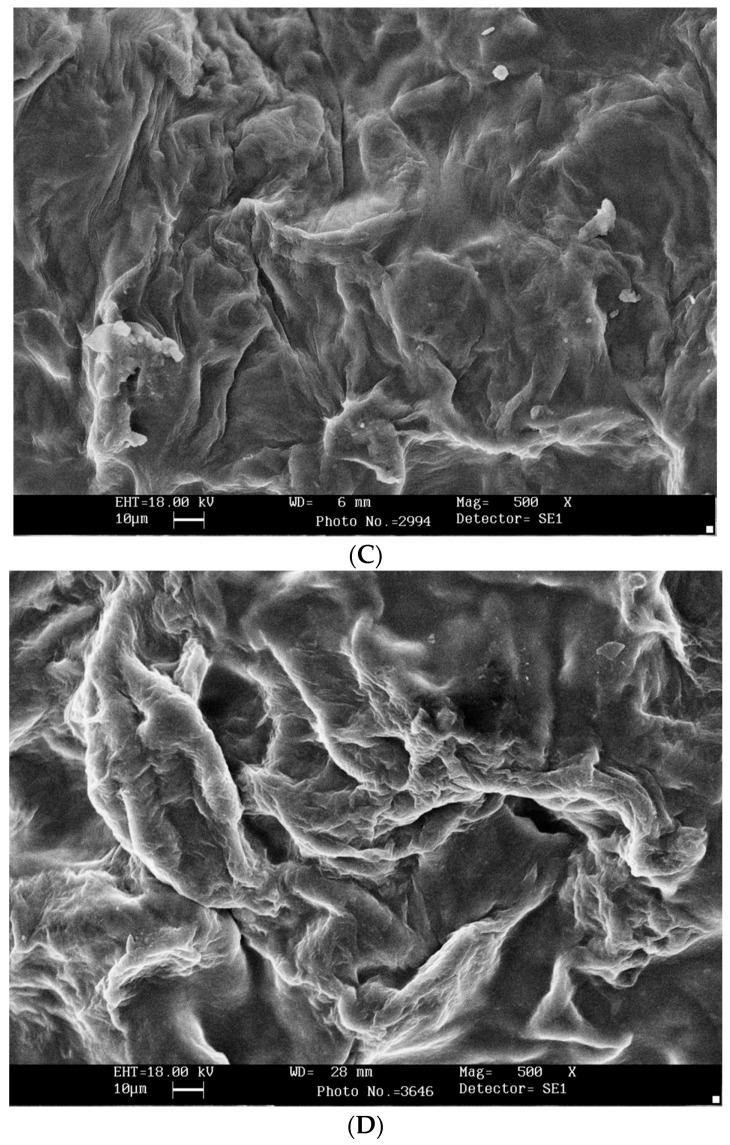
SEM images of surface of convectively dried banana slices pretreated with (**A**) ultrasonic power of 0 W–sample mass to coating solution mass ratio of 1:2, (**B**) ultrasonic power of 1000 W–sample mass to coating solution mass ratio of 1:2, (**C**) ultrasonic power of 0 W–sample mass to coating solution mass ratio of 1:4, and (**D**) ultrasonic power of 1000 W–sample mass to coating solution mass ratio of 1:4.

As Figure 8 depicts, the SEM images were prepared to compare the effect of different ultrasonic powers and BS:CS ratios on the microscopic structures of the dried banana slices. The comparison of the microscopic images of the dried banana slices in 0 W-1:2 (Figure 8A) and 0 W-1:4 (Figure 8C) treatments indicates that by changing the BS:CS ratio from 1:2 to 1:4, the CMC layer on the sample surface pretreated by 0 W-1:4 (Figure 8C) becomes more uniform. By comparing the microscopic images in 1000 W-1:2 (Figure 8B) and 1000 W-1:4 (Figure 8D), it is again observed that by the BS:CS ratio changing from 1:2 to 1:4, the CMC layer on the surface of the banana slices obtained from the treatment of 1000 W-1:4 (Figure 8D) is more uniform. The presence of a more uniform and smoother CMC layer on the surface of the convectively dried banana slices with increasing the sample to CMC solution from 1:1 to 1:2 was reported in a study conducted by Nadery Dehsheikh and Taghian Dinani [62].

To investigate the effect of the ultrasonic process on the microscopic structure of banana slices, a comparison of the two treatments of 1000 W-1:2 (Figure 8B) and 0 W-1:2 (Figure 8A) demonstrates that the CMC layer on the surface of the dried banana slices after the 1000 W-1:2 pretreatments (Figure 8B) is more uniform. The same result is obtained by comparing two treatments of 1000 W-1:4 (Figure 8D) and 0 W-1:4 (Figure 8C), which shows that using the ultrasound process, the coating process of the banana slices resulted in a more uniform CMC layer on the sample surface pretreated by 1000 W-1:4 (Figure 8D). Interestingly, in this situation, along with the uniform CMC layer formation on the sample surface, considerable porosity inside the dried sample is also maintained in these treatments (the cross-sectional SEM images are shown by Nadery Dehsheikh and Taghian Dinani) [77]. In addition, Kaur et al. [78] observed through SEM analysis that ultrasound-assisted pretreatment combined with controlled carbonization of Cavendish banana peel led to the most highly porous microstructure with defined micro-channels. They pointed out that despite the changes in the structure, the collapse of the cellular structure did not take place. Therefore, it can be concluded that a good coating represents the formation of a coating layer with good uniformity on the surface of the sample. In addition, the existence of porosity and tissue channels plays an important role in maintaining and enhancing the dried product quality.

## 4. Conclusions

In this research, for enhancing the quality of dried banana slices and increasing their drying rate, they were treated with carboxymethyl cellulose solution in an ultrasonic system. The findings from this research are as follows:

- The drying rate of treatments was increased with ultrasonic pretreatment, especially at 1000 W, in comparison to treatments without ultrasonic pretreatment. Furthermore, with ultrasonic power increasing from 0 to 1000 W, a significant increase in dependent variables of D_eff_ (*p* ≤ 0.001), WAC (*p* ≤ 0.001), and AA (*p* ≤ 0.001), and a significant decrease in BI (*p* ≤ 0.01) of the dried banana slices was observed.
- By changing the BS:CS ratio from 1:2 to 1:4, a significant increase in the dependent variables of WAC (*p* ≤ 0.01) and AA (*p* ≤ 0.05), and a significant decrease in BI (*p* ≤ 0.01) of the dried banana slices was observed.
- The SEM images showed that by increasing the ultrasonic power from 0 to 1000 W, and by changing the BS:CS ratio from 1:2 to 1:4, a more uniform CMC coating layer was deposited on the surface of the dried banana slices.
- The FT-IR spectra indicated that the functional groups of the dried banana slices were not degraded by ultrasonic pretreatment, and also indicated that various proportions of sample to CMC coating solution resulted in similar FT-IR spectra.

In general, it can be concluded that the CMC coating pretreatment, especially with a BS:CS ratio of 1:4, and with 1000 W ultrasonic power, produces a product with more water absorption, antioxidant capacity, and a lower browning index. Therefore, it is possible to combine these pretreatments with the convective drying method to produce fruit chips as a healthy snack with good quality and nutritional value. However, the initial investment that is required for ultrasonic equipment and the need for a designed production line are important considerations. Due to the importance of coating solution viscosity in coating, drying, ultrasound-based processes, and the product quality, future research should focus on optimizing the CMC viscosity along with different ultrasound parameters. Secondly, applying sensory evaluations and comprehensive nutritional analysis of dried products would provide better insight into the market acceptance.

## Figures and Tables

**Table 1:** ANOVA results of the ultrasonic power and BS:CS ratio variables and their mutual effect.

Change Sources	Degrees of Freedom	Responses			
		D_eff_ (×10^−9^ m^2^/s)	WAC	AA (%)	BI
Ultrasonic power	2	26.16 ***	25.5 ***	19.99 ***	10.25 **
Banana slice mass to the coating solution mass	2	3.01 ^n.s.^	11.1 **	3.91 *	11.54 **
Ultrasonic power × Banana slice mass to the coating solution mass	4	1.06 ^n.s.^	2.85 *	1.04 ^n.s.^	3.24 *

* significant at *p* ≤ 0.05. ** very significant at *p* ≤ 0.01. *** extremely significant at *p* ≤ 0.001. ^n.s.^, not significant.

**Table 2:** The effect of ultrasonic power on the evaluated dependent variables.

Ultrasonic Power (W)	Responses			
	D_eff_ (×10^−9^ m^2^/s)	WAC	AA (%)	BI
0	2.84 ± 0.19 ^c^	0.41 ± 0.02 ^b^	77.63 ± 0.89 ^c^	67.59 ± 3.44 ^a^
500	4.34 ± 0.44 ^b^	0.51 ± 0.04 ^a^	81.60 ± 0.75 ^b^	61.49 ± 1.90 ^b^
1000	5.87 ± 0.28 ^a^	0.68 ± 0.05 ^a^	83.57 ± 0.60 ^a^	56.41 ± 2.26 ^b^

The results are reported as the mean ± standard deviation values. The average values of each variable that are indicated by similar alphabets are insignificant (*p* > 0.05).

**Table 3:** The effect of ratio of the banana slices to the coating solution on the evaluated dependent variables.

Banana Slice Mass to the Coating Solution Mass	Responses			
	D_eff_ (×10^−9^ m^2^/s)	WAC	AA (%)	BI
1:2	3.76 ± 0.58 ^a^	0.43 ± 0.02 ^b^	79.77 ± 1.18 ^b^	67.03 ± 3.62 ^a^
1:3	4.61 ± 0.31 ^a^	0.55 ± 0.05 ^a^	80.64 ± 0.70 ^b^	63.09 ± 2.28 ^a^
1:4	4.67 ± 0.62 ^a^	0.61 ± 0.06 ^a^	82.39 ± 1.28 ^a^	55.37 ± 1.13 ^b^

The results are reported as the mean ± standard deviation values. The average values of each variable that are indicated by similar alphabets are insignificant (*p* > 0.05).

**Table 4:** The correlation coefficients between pairs of the dependent variables.

Adjectives	D_eff_ (×10^−9^ m^2^/s)	WAC	AA (%)	BI
D_eff_ (×10^−9^ m^2^/s)	1			
WAC	0.66 **	1		
AA (%)	0.65 **	0.67 **	1	
BI	−0.50 *	−0.56 **	−0.51 **	1

* Significant at *p* ≤ 0.05. ** Significant at *p* ≤ 0.01.

## Data Availability

The original contributions presented in this study are included in the article material. Further inquiries can be directed to the corresponding author.

## Abbreviations

The following abbreviations are used in this manuscript:

CMC	Carboxymethyl Cellulose
BS:CS	Banana Slice mass to the Coating Solution mass
D_eff_	Effective Water Diffusion Coefficient
WAC	Water Absorption Capacity
AA	Antioxidant Activity
BI	Browning Index
SEM	Scanning Electron Microscopy
FT-IR	Fourier-Transform Infrared Spectroscopy
EHD	Electrohydrodynamic
MC	Moisture Content
MR	Moisture Ratio
DR	Drying Rate
DPPH	2-Diphenyl-1-Picrylhydrazyl
SE	Standard Error

## References

[1] Sulaiman S.F., Yusoff N.A.M., Eldeen I.M., Seow E.M., Sajak A.A.B., Supriatno, Ooi K.L. (2011). Correlation between total phenolic and mineral contents with antioxidant activity of eight Malaysian bananas (*Musa* sp.). J. Food Compos..

[2] Arvanitoyannis I., Mavromatis A. (2009). Banana cultivars, cultivation practices, and physicochemical properties. Crit. Rev. Food Sci. Nutr..

[3] Senadeera W., Bhandari B., Young G., Wijesinghe B. (2005). Modelling dimensional shrinkage of shaped foods in fluidized bed drying. J. Food Process. Preserv..

[4] Waliszewski K.N., Texon N.I., Salgado M.A., Garcia M.A. (1997). Mass transfer in banana chips during osmotic dehydration. Dry. Technol..

[5] Abrahão F.R., Gomes Correa J.L., Sousa A.D.B.M., Silveira P.G., Nepomuceno da Cunha R. (2024). Effect of ultrasound and osmotic dehydration as pretreatments on the infrared drying of banana slices. Food Technol. Biotechnol..

[6] Jiang H., Zhang M., Mujumdar A.S., Lim R.X. (2014). Changes of microwave structure/dielectric properties during banana microwave freeze-drying process. Int. J. Food Sci..

[7] Salunke S., Yadav K.C., Kadam S. (2019). Effect of microwave drying on drying and quality characteristics of banana chips. J. Pharmacogn. Phytochem..

[8] Tavakolipour H., Zirgani L. (2014). Combined hot air–microwave drying methods in banana chips production. Am.-Eurasian J. Agric. Env. Sci..

[9] Milani A., Jouki M., Rabbani M. (2020). Production and characterization of freeze-dried banana slices pretreated with ascorbic acid and quince seed mucilage: Physical and functional properties. Food Sci. Nutr..

[10] Pan Z., Shih C., Mchugh T.H., Hirschberg E. (2008). Study of banana dehydration using sequential infrared radiation and freeze-drying (SIRFD) method: Quality and drying characteristics. LWT-Food Sci. Technol..

[11] Taskin O. (2025). Study on the vacuum freeze-drying of banana and impact on powder properties. Case Stud. Therm. Eng..

[12] Figiel A. (2010). Drying kinetics and quality of Beetroots dehydrated by combination of convective and vacuum-microwave methods. J. Food Eng..

[13] Lin T., Durance T., Scaman C. (1998). Characterization of vacuum microwave, air and freeze dried carrot slices. Food Res. Int..

[14] Maroulis Z.B., Tsami E., Marinos-Kouris D., Saravacos G.D. (1988). Application of the GAB model to the sorption isotherms of dried fruits. J. Food Eng..

[15] Rodríguez O., Santacatalina J.V., Simal S., García-Pérez J., Femenia A., Rosselló C. (2014). Influence of power ultrasound application on drying kinetics of apple and its antioxidant and microstructural properties. J. Food Eng..

[16] Taghian Dinani S., Hamdami N., Shahedi M., Keramat J. (2013). Optimization of carboxymethyl cellulose and calcium chloride dip-coating on mushroom slices prior to hot air drying using response surface methodology. J. Food Process. Preserv..

[17] Ojha K., Tiwari B., O’Donnell C. (2018). Effect of Ultrasound Technology on Food and Nutritional Quality. Adv. Food Nutr. Res..

[18] Soria A., Villamiel M. (2010). Effect of ultrasound on the technological properties and bioactivity of food: A review. Trends Food Sci. Technol..

[19] Patist A., Bates D. (2008). Ultrasonic innovations in the food industry: From the laboratory to commercial production. Innov. Food Sci. Emerg. Technol..

[20] Kilmanoğlu H., Hoşoğlu M., Güneşer O., Yüceer Y. (2021). Optimization of pretreatment and enzymatic hydrolysis conditions of tomato pomace for production of alcohols and esters by *Kluyveromyces marxianus*. LWT.

[21] Ren F., Perussello C., Zhang Z., Kerry J., Tiwari B. (2018). Impact of ultrasound and blanching on functional properties of hot-air dried and freeze dried onions. LWT.

[22] Fernandes F., Linhares F., Rodrigues S. (2008). Ultrasound as pre-treatment for drying of pineapple. Ultrason. Sonochem..

[23] García-Pérez J., Cárcel J., Benedito J., Mulet A. (2007). Power ultrasound mass transfer enhancement on food drying. Food Bioprod. Process..

[24] Romero J.C.A., Yépez V.B.D. (2015). Ultrasound as pretreatment to convective drying of Andean blackberry (*Rubus glaucus Benth*). Ultrason. Sonochem..

[25] Horuz E., Jaafar H., Maskan M. (2017). Ultrasonication as pretreatment for drying of tomato slices in a hot air–microwave hybrid oven. Dry. Technol..

[26] Rodríguez Ó., Eim V., Rosselló C., Femenia A., Cárcel J.A., Simal S. (2018). Application of power ultrasound on the convective drying of fruits and vegetables: Effects on quality. J. Sci. Food Agric..

[27] Nowacka M., Wiktor A., Sledz M., Jurek N., Witrowa-Rajchert D. (2012). Drying of ultrasound pretreated apple and its selected physical properties. J. Food Eng..

[28] Mieszczakowska-Frąc M., Dyki B., Konopacka D. (2016). Effects of ultrasound on polyphenol retention in apples after the application of predrying treatments in liquid medium. Food Bioprocess Technol..

[29] Zhou S., Chen W., Chitrakar B., Fan K. (2024). Ultrasound technology for enhancing drying efficiency and quality of fruits and vegetables: A review. Food Bioprocess Technol..

[30] Zhang M., Ding C., Yang J., Lin S., Chen L., Huang L. (2016). Study of interaction between water-soluble collagen and carboxymethyl cellulose in neutral aqueous solution. Carbohydr. Polym..

[31] Kaushal M., Sharma P.C., Sharma R. (2011). Formulation and acceptability of foam mat dried seabuckthorn (*Hippophae salicifolia*) leather. J. Food Sci. Technol..

[32] Rajabi F., Karimi S., Abbasi H., Layeghinia N. (2025). Influence of edible coatings pretreatment on the performance of microwave and combined microwave-hot air drying of kiwifruit. Food Bioprod. Process..

[33] Zang Z., Huang X., Ma G., Wan F., Xu Y., Zhao Q., Wu B., Lu H., Liu Z. (2025). Novel edible coatings pretreatment for enhancing drying performance and physicochemical properties of cherry fruits during multi-frequency ultrasonic vacuum far infrared radiation—Radio frequency vacuum segmented combination drying. Ultrason. Sonochem..

[34] Salehi F., Inanloodoghouz M. (2023). Effects of gum-based coatings combined with ultrasonic pretreatment before drying on quality of sour cherries. Ultrason. Sonochem..

[35] Zang Z., Wan F., Xu Y., Wu B., Huang X. (2024). Effect of ultrasound combined with chemical pretreatment as an innovative non-thermal technology on the drying process, quality properties, and texture of cherry subjected to radio frequency vacuum drying. Ultrason. Sonochem..

[36] Demirel D., Turhan M. (2003). Air-drying behavior of dwarf Cavendish and Gros Michel banana slices. J. Food Eng..

[37] Azoubel P.M., Baima M.D.A.M., da Rocha Amorim M., Oliveira S.S.B. (2010). Effect of ultrasound on banana cv Pacovan drying kinetics. J. Food Eng..

[38] Taghian Dinani S., Havet M., Hamdami N., Shahedi M. (2014). Drying of mushroom slices using hot air combined with an electrohydrodynamic (EHD) drying system. Dry. Technol..

[39] Bagheri N., Taghian Dinani S. (2019). Investigation of ultrasound-assisted convective drying process on quality characteristics and drying kinetics of zucchini slices. Heat Mass Transf..

[40] Dehghannya J., Hosseinlar S.-H., Heshmati M. (2018). Multi-stage continuous and intermittent microwave drying of quince fruit coupled with osmotic dehydration and low temperature hot air drying. Innov. Food Sci. Emerg. Technol..

[41] Lewicki P. (1998). Some remarks on rehydration of dried foods. J. Food Eng..

[42] Haji Heidari S., Taghian Dinani S. (2018). The study of ultrasound-assisted enzymatic extraction of oil from peanut seeds using response surface methodology. Eur. J. Lipid Sci. Technol..

[43] Palou E., López-Malo A., Barbosa-Cánovas G., Welti-Chanes J., Swanson B. (1999). Polyphenoloxidase activity and color of blanched and high hydrostatic pressure treated banana puree. J. Food Sci..

[44] Doymaz I., Göl E. (2011). Convective drying characteristics of eggplant slices. J. Food Process. Eng..

[45] Sun Y., Ma G., Ye X., Kakuda Y., Meng R. (2010). Stability of all-trans-β-carotene under ultrasound treatment in a model system: Effects of different factors, kinetics and newly formed compounds. Ultrason. Sonochem..

[46] Jambrak A., Mason T., Paniwny L., Lelas V. (2007). Accelerated drying of button mushrooms, Brussels sprouts and cauliflower by applying power ultrasound and its rehydration properties. J. Food Eng..

[47] Puig A., Perez-Munuera I., Carcel J.A., Hernando I., Garcia-Perez J.V. (2012). Moisture loss kinetics and microstructural changes in eggplant (*Solanum melongena* L.) during conventional and ultrasonically assisted convective drying. Food Bioprod. Process..

[48] Yu F., Li Y., Wu Z., Wang X., Wan N., Yang M. (2020). Dehydration of wolfberry fruit using pulsed vacuum drying combined with carboxymethyl cellulose coating pretreatment. LWT.

[49] Golafshani E., Jafari M., Kashanynejad M., Bezraghi Tosi S. (2016). The effect of coating with carrageenan and carboxymethylcellulose on the process of osmosis on drying time and water reappropriation in Lebanese yellow apple. J. Food Ind. Res..

[50] An N.-N., Shang N., Lv W.-Q., Li D., Wang L.-J., Wang Y. (2022). Effects of carboxymethyl cellulose/pectin coating combined with ultrasound pretreatment before drying on quality of turmeric (*Curcuma longa* L.). Int. J. Biol. Macromol..

[51] Ortuño C., Pérez-Munuera I., Puig A., Riera E., García-Pérez J. (2010). Influence of power ultrasound application on mass transport and microstructure of orange peel during hot air drying. Phys. Procedia.

[52] Salehi F., Inanloodoghouz M., Karami M. (2023). Rheological properties of carboxymethyl cellulose (CMC) solution: Impact of high intensity ultrasound. Ultrason. Sonochem..

[53] Wang Y., Zhang M., Mujumdar A., Mothibe K., Roknul Azam S. (2013). Study of drying uniformity in pulsed spouted microwave–vacuum drying of stem lettuce slices with regard to product quality. Dry. Technol..

[54] Garcia-Perez J., Puig A., Perez-Munuera I., Carcel J., Riera E. Kinetic and microstructural changes induced by power ultrasound application on convective drying of eggplant. Proceedings of the 20th International Congress on Acoustics, 20th International Congress on Acoustics.

[55] Jadhav D.B., Visavale G.L., Sutar N., Annapure U.S., Thorat B.N. (2010). Studies on solar cabinet drying of green peas (*Pisum sativum*). Dry. Technol..

[56] Eshraghi E., Maghsoudlo Y., Kashani Nejad M., Bezraghi S., Aalami M. (2011). The effect of ultrasound pretreatment on drying sliced kiwi. Iran. Food Sci. Technol..

[57] Khin M., Zhou W., Perera O. (2007). Impact of process conditions and coatings on the dehydration efficiency and cellular structure of apple tissue during osmotic dehydration. J. Food Eng..

[58] Askari G., Emam-Djomeh Z., Mousavi M. (2006). Effects of combined coating and microwave assisted hot-air drying on the texture, microstructure and rehydration characteristics of apple slices. Food Sci. Technol. Int..

[59] Bassey E., Cheng J., Sun D. (2021). Novel nonthermal and thermal pretreatments for enhancing drying performance and improving quality of fruits and vegetables. Trends Food Sci. Technol..

[60] Lin D., Zhao Y. (2007). Innovation the development and application of edible coating for fresh and minimally processed fruits and vegetables. Compr. Rev. Food Sci. Food Saf..

[61] Lobo F., Nascimento M., Domingues J., Falcão DQ H.F., de Lima Araujo K. (2017). Foam mat drying of Tommy Atkins mango: Effects of air temperature and concentrations of soy lecithin and carboxymethylcellulose on phenolic composition, mangiferin, and antioxidant capacity. Food Chem..

[62] Nadery Dehsheikh F., Taghian Dinani S. (2020). Influence of coating pretreatment with carboxymethyl with carboxymethyl cellulose in an electrohydrodynamic system on convective drying of banana slices. J. Food Process. Eng..

[63] Sakooei-Vayghan R., Peighambardoust S., Hesari J., Peressini D. (2020). Effects of osmotic dehydration (with and without sonication) and pectin-based coating pretreatments on functional properties and color of hot-air dried apricot cubes. Food Chem..

[64] Delgado-Vargas F., Paredes-López O. (2002). Natural Colorants for Food and Nutraceutical Uses.

[65] Fu Y.H., Zhang Y., Wang F., Zhao L., Shen G.B., Zhu X.Q. (2023). Quantitative evaluation of the actual hydrogen atom donating activities of O–H bonds in phenols: Structure–activity relationship. RSC Adv..

[66] Biswas R., Sayem A.S.M., Alam M., Sun D.W., Hossain M.A. (2025). Combined ultrasound and osmotic pretreatment as innovative preservation strategies for enhancing the quality of dried mango slices. LWT.

[67] Shahram H., Taghian Dinani S., Amouheydari M. (2019). Effects of pectinase concentration, ultrasonic time, and pH of an ultrasonic-assisted enzymatic process on extraction of phenolic compounds from orange processing waste. J. Food Meas. Charact..

[68] Deng Y., Zhao Y. (2008). Effect of pulsed vacuum and ultrasound osmopretreatments on glass transition temperature, texture, microstructure and calcium penetration of dried apples (Fuji). LWT-Food Sci. Technol..

[69] Garcia-Noguera J., Olive F., Weller C., Rodrigues S., Fernandes F. (2014). Effect of ultrasonic and osmotic dehydration pre-treatments on the colour of freeze dried strawberries. J. Food Sci. Technol..

[70] Deng L.-Z., Mujumdar A.S., Zhang Q., Yang X.-H., Wang J., Zheng Z.-A., Gao Z.-J., Xiao H.-W. (2017). Chemical and physical pretreatments of fruits and vegetables: Effects on drying characteristics and quality attributes. Crit. Rev. Food Sci. Nutr..

[71] Hosseinpour S., Rafiee S., Mohtasebi S., Aghbashlo M. (2013). Application of computer vision technique for on-line monitoring of shrimp color changes during drying. J. Food Eng..

[72] Ramachandran M., Bansal S., Raichurkar P. (2016). Experimental study of bamboo using banana and linen fibre reinforced polymeric composites. Perspect. Sci..

[73] Rahimpour S., Taghian Dinani S. (2018). Lycopene extraction from tomato processing waste using ultrasound and cell-wall degrading enzymes. J. Food Meas. Charact..

[74] Pelissari F., Andrade-Mahecha M., Sobral P.J., Menegalli F.C. (2013). Comparative study on the properties of flour and starch films of plantain bananas (*Musa paradisiaca*). Food Hydrocoll..

[75] Khalili F., Taghian Dinani S. (2018). Extraction of phenolic compounds from olive-waste cake using ultrasonic process. J. Food Meas. Charact..

[76] Wang H., Han P., Zhao Y., Lu L., Qi W., Zhao K., Shu Y., Zhang Z. (2025). Preparation and characteristics of carboxymethyl cellulose-based films embedding cinnamon essential oil and their application on mutton preservation. Front. Nutr..

[77] Nadery Dehsheikh F., Taghian Dinani S. (2019). Coating pretreatment of banana slices using carboxymethyl cellulose in an ultrasonic system before convective drying. Ultrason. Sonochem..

[78] Kaur M., Modi V., Sharma H. (2022). Evaluation of ultrasonication and carbonation-ultrasonication assisted convective drying techniques for enhancing the drying rates and quality parameters of ripe and raw banana (*Musa*) peel. J. Food Sci. Technol..

